# Emergence of Undetectable Malaria Parasites: A Threat under the Radar amid the COVID-19 Pandemic?

**DOI:** 10.4269/ajtmh.20-0467

**Published:** 2020-06-16

**Authors:** Khalid B. Beshir, Lynn Grignard, Khalid Hajissa, Abdulrahman Mohammed, Awolkhier M. Nurhussein, Deus S. Ishengoma, Inke Nadia D. Lubis, Chris J. Drakeley, Colin J. Sutherland

**Affiliations:** 1 Faculty of Infectious and Tropical Diseases, London School of Hygiene and Tropical Medicine, London, United Kingdom;; 2 Department of Medical Microbiology and Parasitology, School of Medical Sciences, Universiti Sains Malaysia, Kelantan, Malaysia;; 3 National Public Health Reference Laboratory, Ministry of Health, Mogadishu, Somalia;; 4 University Hospitals Coventry and Warwickshire, NHS Trust, Coventry, United Kingdom;; 5 National Institute for Medical Research, Dar es Salaam, Tanzania;; 6 Faculty of Medicine, Universitas Sumatera Utara, Medan, Indonesia

## Abstract

Rapid diagnostic tests (RDTs) play a critical role in malaria diagnosis and control. The emergence of *Plasmodium falciparum* parasites that can evade detection by RDTs threatens control and elimination efforts. These parasites lack or have altered genes encoding histidine-rich proteins (HRPs) 2 and 3, the antigens recognized by HRP2-based RDTs. Surveillance of such parasites is dependent on identifying false-negative RDT results among suspected malaria cases, a task made more challenging during the current pandemic because of the overlap of symptoms between malaria and COVID-19, particularly in areas of low malaria transmission. Here, we share our perspective on the emergence of *P. falciparum* parasites lacking HRP2 and HRP3, and the surveillance needed to identify them amid the COVID-19 pandemic.

## INTRODUCTION

A previously unknown coronavirus was identified as the causative agent of serious respiratory illness in January 2020 in Wuhan, China. The WHO named the virus SARS-CoV-2, and the disease was named COVID-19.^1^ World leaders have mobilized their resources to fight the pandemic, which has caused more than 7 million cases and more than 400,000 deaths worldwide as of June 8, 2020. In Africa, more than 200,000 COVID-19 cases and 5,000 deaths were reported in 57 countries as of that date. Whereas the world is focused on COVID-19 and resources are diverted to contain the pandemic, other infectious diseases such as malaria, tuberculosis, and HIV/AIDS continue to affect millions, particularly in Africa.

## MALARIA—ONE OF THE LEADING CAUSES OF DEATH IN AFRICA

In 2019, the WHO reported an estimated 228 million cases of malaria worldwide, mostly in Africa (213 million cases), and 405,000 deaths, most of which were in the WHO African region (94%).^2^ Progress has been made in reducing the number of cases and deaths associated with effective vector control, the deployment of rapid diagnostic tests (RDTs), and effective, accessible treatment. The COVID-19 pandemic threatens to reverse these achievements because of erosion of health systems and disruption of malaria control and elimination programs. The WHO considers a worst-case scenario, with distribution of insecticide-treated nets for 2020 canceled and a reduction in effective malaria treatment by 75%, leading to a 23% increase in malaria cases and a 102% increase in deaths.^3^ Iran, for example, having reported zero indigenous malaria cases for the first time in 2018, is severely impacted across its entire health system by the pandemic, with high numbers of confirmed COVID-19 cases and deaths posing a threat to the sustainability of its malaria-free status.

## MALARIA DIAGNOSIS

Since 2010, WHO malaria treatment guidelines have included recommendations to test all suspected cases by microscopy or RDTs. Both can provide definitive malaria diagnosis within minutes. Because accurate microscopic examination requires expertise and is not always available in remote areas, RDTs are increasingly used, particularly at the community level and in primary healthcare facilities. Histidine-rich protein 2 (HRP2)–based RDTs detect only *Plasmodium falciparum*, whereas alternatives that detect lactate dehydrogenase (LDH) or aldolase are used for *P. falciparum* only (Pf-LDH), *Plasmodium vivax* only (Pv-LDH), all species other than *P. falciparum* (Pvom-pLDH), or all *Plasmodium* species (pan-LDH; aldolase). Rapid diagnostic tests that detect HRP2 are the most widely used tests against *P. falciparum*, as RDTs that detect LDH and aldolase are less sensitive and more susceptible to degradation from heat and humidity during transport and storage.^4,5^ Some RDTs in use detect a combination of antigens such as Pf-HRP2/Pf-LDH, Pf-HRP2/pan-LDH, or Pf-HRP2/Pv-LDH (Table 1).

**Table 1:** COVID-19 cases and malaria diagnosis method (in community setting and primary health facilities) in selected countries where *hrp2*/3 deletion has been reported

Country	COVID-19 cases*	Malaria diagnosis method^2^	Estimated *hrp2/3* gene deletion prevalence†	Reference for *hrp2/3* deletion
Eritrea	41	Pan-LDH RDT‡	62%	7,11
Ethiopia	> 500	Pf-HRP2/Pv-LDH RDT	Not known	11,15
Kenya	> 1,000	Pf-HRP2 RDT	< 5%	8
Somalia	> 2,000	Pf-HRP2/Pan-LDH RDT	Not known	11
Sudan	> 3,000	Pf-HRP2/Pv-LDH RDT	3–5%	11,16
South Sudan	> 400	Pf-HRP2 RDT	5–10%	11
Tanzania	> 500	Pf-HRP2/Pan-LDH RDT	1%	10, 11

LDH = lactate dehydrogenase; RDT = rapid diagnostic test.

* As of June 8, 2020.

† Based on sporadic evidence.

‡ Recently changed from Pf-HRP2/Pv-LDH RDT to Pan-LDH RDT = *Plasmodium*-lactate dehydrogenase; Pv-LDH = *P. vivax*-LDH.

## THE EMERGENCE OF “DIAGNOSTIC RESISTANCE” IN MALARIA PARASITES

“Diagnostic resistance” is due to the emergence of *P. falciparum* strains that escape RDT detection because of deletion of genes encoding HRP2 and HRP3, the antigens recognized by the most commonly used RDTs. *Plasmodium falciparum* parasites with these deletions first emerged in South America^6^ and were subsequently reported in Southeast Asia and Africa.^7^ Our team has described their circulation in Kenya,^8^ Tanzania,^9^ and Uganda.^10^ Our most recent data from travelers returning to the United Kingdom with malaria provide evidence for such parasites in Somalia and South Sudan, and suggests they also circulate in Eritrea, Ethiopia, Kenya, Sudan, and Uganda.^11^ Countries with a high prevalence of these deletions, such as Peru and Eritrea, have already removed HRP2-based RDTs from their diagnostic policies, but alternatives are scarce, more expensive, and require compromises in sensitivity.

## IS IT MALARIA OR COVID-19?

The most common symptoms of SARS-CoV-2 infection have been reported as fever, cough, and shortness of breath.^12^ The WHO defines a suspected COVID-19 case as a patient with acute respiratory illness (fever and at least one sign/symptom of respiratory disease such as cough) in the absence of an alternative diagnosis that fully explains the clinical presentation.^12^ However, not all virus-positive COVID-19 patients exhibit respiratory symptoms, and the spectrum of presentation is now understood to be broad. This is further complicated in malaria-endemic countries, where fever is also the main symptom of malaria. If a patient shows symptoms common to both COVID-19 and malaria, an obvious solution would be to rule out malaria infection using an RDT, a strategy used to rapidly triage malaria patients during the West African Ebola virus outbreak in 2016–2017.^13,14^ However, as described earlier, the results of HRP2-RDTs are no longer completely reliable in some parts of the world, particularly in low transmission settings.

## WHAT NEEDS TO BE DONE?

The WHO has recently published a document entitled “Tailoring malaria interventions in the COVID-19 response.”^17^ The document emphasizes the continued use of RDTs for suspected malaria cases after ruling out other possible causes of fever as per national guidelines. The document recommends that, under special circumstances, to overcome challenges that have arisen because of the COVID-19 pandemic such as supply chain disruption for RDTs, absenteeism of health workers, shortage of personal protective equipment, and the ambiguity of fever symptoms, a malaria diagnosis be considered for all fever cases in endemic countries. This approach, under the special circumstances mentioned earlier, could ensure treatment of RDT-negative malaria patients and, at the population level, potentially curtail the spread of *P. falciparum* parasites carrying the *hrp2/3* deletion. Although efforts to control *hrp2/3*-deleted parasites benefit from the presumptive treatment of malaria cases, a long-term sustainable solution should be in place to ensure the continued surveillance and control of such parasite strains beyond the COVID-19 pandemic.

The safety of patients must be taken into consideration, especially in remote primary health centers, which are heavily dependent on RDTs for malaria diagnosis because of the absence of microscopy. Careful management will be required when treating COVID-19–suspected patients with artemisinin combination therapies (ACTs), which are known to cause corrected QT interval prolongation in some patients.^18–21^ Despite the lack of an established effective treatment for COVID-19, drugs such as chloroquine, hydroxychloroquine, and azithromycin have been widely used,^22^ and these can also cause corrected QT interval prolongation, even at doses regarded as safe.^20,23^ Administration of these drugs to COVID-19 patients who have already received a presumptive dose of ACTs because of febrile presentation may then lead to cardiac complications.

The WHO has called for ministries of health and national malaria control programs to ensure that malaria control efforts are not hampered while tackling the COVID-19 response.^17^ Malaria parasites with *hrp2/3* deletions are now known to be present in 31 countries, but the epidemiology of this problem is not sufficiently well documented to permit a targeted response.^24^ In low malaria transmission areas, where age-groups with symptoms of malaria and COVID-19 are expected to overlap, investigation of fevers is challenging because of the possibility of *hrp2/3* deletions, particularly if only HRP2-based RDTs are used for diagnosis. An alternative approach is to conduct systematic surveillance of *hrp2/3* deletions in priority countries using a non–HRP2-based RDT. A template protocol developed by the WHO is now available to guide the surveillance of *hrp2/3* deletions.^25^ The protocol recommends the use of diagnostics recognizing Pf-HRP2 and Pf-LDH or microscopy to identify suspected *hrp2/3* deletions.

The diagnostic challenges caused by the COVID-19 pandemic offer an ideal opportunity to scale up the surveillance for *hrp2/3* diagnostic resistance in *P. falciparum*, initially by rapid adoption of the WHO surveillance protocol. A variety of RDTs for SARS-CoV-2 have emerged, and a similar effort against malaria could provide an alternative to HRP2-based RDT in the medium term, particularly if they can be developed in endemic settings. Efforts should focus on antigens that are more sensitive and heat stable than the non-HRP2 tests currently available.
